# Nearby Nature ‘Buffers’ the Effect of Low Social Connectedness on Adult Subjective Wellbeing over the Last 7 Days

**DOI:** 10.3390/ijerph15061238

**Published:** 2018-06-12

**Authors:** Benjamin D. S. Cartwright, Mathew P. White, Theodore J. Clitherow

**Affiliations:** European Centre for Environment & Human Health (ECEHH), University of Exeter Medical School, Knowledge Spa, Royal Cornwall Hospital Treliske, Truro, Cornwall TR1 3HD, UK; bdsc201@exeter.ac.uk (B.D.S.C.); tc446@exeter.ac.uk (T.J.C.)

**Keywords:** natural environments, perceived nature, social connectedness, subjective wellbeing

## Abstract

Subjective wellbeing (SWB) is positively associated with both social connectedness and contact with natural environments. However, few studies have explored how these two predictors of SWB might interact. The current work hypothesised that high levels of nature exposure might mitigate (or buffer) any negative effects of a lack of recent social connectedness on wellbeing, by offering an alternative way for individuals to connect with the world around them. Results are based on data from 359 individuals who responded to an online survey in the UK. Measures of SWB, social connectedness and nature visit frequency all focused on the last seven days, and nearby nature was assessed in terms of the view from home and surrounding area. Regression models predicting SWB used interaction terms to test the buffering hypothesis, and controlled for sociodemographic and personality variables. Supporting predictions, social connectedness, nearby nature, and nature visit frequency were all positively associated with SWB. Partially supporting our buffering hypothesis, nearby nature, but not nature visit frequency, moderated the relationship between social connectedness and SWB. People with poor social connectedness still showed high levels of wellbeing if they reported high levels of nearby nature, and there was a lower likelihood of levels of wellbeing associated with depression among those with poor social connectedness if they had high nearby nature. Results confirmed the importance of nature exposure for wellbeing in itself, and highlighted its potential role in offering socially isolated individuals a way of satisfying the need to feel connected.

## 1. Introduction

### 1.1. Overview

Achieving positive subjective wellbeing and reducing the prevalence and challenges presented by mental health disorders is attracting ever-increasing interest from policy-makers, practitioners and the general public. Promoting good mental health is, for instance, a central objective of the World Health Organisation’s (WHO) Global Mental Health Action Plan, and included in the UN’s Sustainable Development Goals [1,2]. Further, there is also growing recognition that mental health is not just about the absence of mental conditions such as anxiety and depression, but also the promotion of subjective wellbeing (SWB, i.e., how people think and feel about their lives; [3]). The determinants of good mental health and SWB are complex and varied, and include genetic dispositions, environmental factors, life conditions and opportunities, lifestyle and other behaviours, personality and patterns of thoughts [4,5,6,7,8,9]. Two antecedents which have received a considerable amount of research attention in recent years are: (a) social connectedness and its converse, social isolation [10]; and (b) contact with natural environments, especially among a growing urban population [11,12]. By and large, contact with other people and contact with natural environments are both associated with better mental health and SWB.

Although most research has considered these two predictors largely independently, a growing area of research has suggested that social connectedness may mediate the relationship between natural environments and mental health and SWB by providing a physical opportunity for people to meet and connect with others [13,14]. Evidence supporting this hypothesis has, however, been highly equivocal with many studies failing to find any evidence of such a mediating process, or finding that the existence of mediation is dependent on specific characteristics of nature exposure and on the social contexts involved [15,16,17,18,19,20,21]. The current research extends previous investigations into the relationship between social connectedness, nature exposure and mental health and wellbeing by adopting a quite different perspective. Building on a psychological needs framework we suggest that contact with either (a) people, or (b) nature, may be satisfying the same underlying need, the need for connectedness/to belong [22], and thus may be somewhat substitutable. Such an approach would predict, for instance, that even if an individual had relatively little social contact, they might still have relatively high wellbeing if they maintained good contact with nature. Put differently, might contact with natural environments “buffer” the traditionally negative association between poor social connectedness and wellbeing? Before we explain how we tested this possibility, we provide greater detail about the theoretical and empirical context of our investigation.

### 1.2. Social Connectedness and Mental Health and Wellbeing

Of the numerous factors influencing mental health and SWB, considerable evidence shows social experiences to be fundamental [10]. As highly social animals, it has been argued that humans have a strong innate need to affiliate with conspecifics and to feel as if they belong to a social group [22]. Subjective perceptions and feelings about one’s social experiences (i.e., perceived social connectedness), as well as objective social events and situations (i.e., actual social connectedness), have both been found to be distinctly important in mental health and SWB [23,24]. Intriguingly, while people who are lacking in objective social connectedness do often report feeling lonely, perceived and actual social connectedness do not always correlate strongly and may sometimes be completely uncorrelated, with factors such as personality, gender, culture, age, genetics, and non-social experiences being influential in one’s perceptions of their social connectedness [25,26,27].

In the current research we focused on actual social connectedness (hereafter referred to just as social connectedness), as operationalised by the number of social contacts in the last week. Social connectedness and the social support that emerges from it provide the individual with core psychological benefits such as sense of meaning, self-esteem, sense of belonging and companionship [28]. Concordantly, they have consistently been shown to be positively associated with SWB, and negatively associated with depression and other mental health disorders [10,24,27,28,29,30,31,32,33]. Face-to-face contact with friends and relatives is a key element of social connectedness and is generally correspondingly associated with mental health and SWB [27,34,35,36,37,38,39,40,41,42]. Although the increasing prevalence of electronically-based social interaction may aid connectedness with social ties, direct contact with friends and family is more important [42]. A sizable portion of society is lacking in this form of contact, potentially impacting public wellbeing [43]. Current trends in developed countries, including decreasing family size, increasing divorce rates, geographical dispersal for employment, and ageing populations, all threaten to diminish the quantity and quality of contact with close friends and relatives in the future, and as such may present a barrier to SWB.

### 1.3. Nature Exposure and Mental Health and Wellbeing

Discussions concerning public health and wellbeing are increasingly recognising that contact with natural features and environments (hereafter referred to as nature exposure) has potentially significant salutogenic (i.e., health-promoting) effects on SWB [11,44]. Nature exposure in day-to-day life may occur intentionally (“experiencing or being in nature through direct intention”), incidentally (“experiencing nature as a by-product of another activity”), or indirectly (“experiencing nature while not being physically present in it”), with each form providing different profiles of psychological effects, and varying in intensity [11]. Epidemiological studies have observed reduced rates of depression, depressive symptomology and antidepressant prescription among people who had more nature exposure (often inferred from the level of greenspace in the area surrounding the home—or ‘nearby nature’—as well as through metrics including accessibility and usage patterns of local areas of nature), along with reduced stress levels, lower prevalence of anxiety and general psychological distress, and improved hedonic and eudaimonic wellbeing [19,45,46,47,48,49,50,51]. Longitudinal panel analysis has also demonstrated that the positive psychological effects of moving to a more natural area remain elevated for some years after a move [52,53]. To the extent that social connectedness is associated with many of these same psychophysiological mechanisms and epidemiological outcomes, it is feasible that the buffering or restorative effects of nature exposure may alleviate the negative influence of social isolation on SWB.

### 1.4. Commonalities in the Relationships between Social and Nature Exposure and Wellbeing

As well as sharing outcomes in mental health and SWB, social and environmental contexts are implicated in a number of the same processes and mechanisms that may underlie these outcomes. These include: psychophysiological stress [54,55]; attention [56,57]; cognitive functioning [58,59]; age-related cognitive decline [60,61]; rumination [62,63]; pain relief and pain tolerance [64,65]; pleasure, mood and affect [49,66,67,68]; self-esteem [69,70]; anger and aggression [71,72]; physical health and exercise [20,44,73]; and, sense of belonging [14,74], among others.

Another interesting similarity between the effects of nature exposure and social connectedness is that both lead to feelings of connection or relatedness, with such feelings speculated to be fundamental human needs that evolved due to the adaptive benefits of being connected with other people and with stable, resource-rich ecologies [22,23,75,76]. There also appears to be some overlap between social and nature connectedness in terms of the behaviours they elicit: experimental studies have found that exposing subjects to natural imagery, features and settings elicits more pro-social values and behaviours than less natural conditions, with this relationship being demonstrated in some cases to be mediated by the feeling of connection with nature [77,78,79,80]. Conversely, individuals with more interdependent self-construals, more pro-social personality traits, and a tendency to be empathetic, report greater feelings of connection with nature and stronger pro-environmental concerns, values and behaviours [81,82,83,84,85]. In short, these studies imply that connectedness in one domain (i.e., social or environmental) may elicit a broader feeling of connection in general, resulting in positive outcomes for others (e.g., pro-social or pro-environmental behaviours). This hypothesis is related to the debate and research concerning the domain- or needs-based structure of human wellbeing, as in Maslow’s notion of the Hierarchy of Needs, and Ryan and Deci’s Self-Determination Theory [75,86]. Firstly, it supposes that nature’s positive mental wellbeing outcomes may occur due to the meeting of connectedness/relatedness needs by nature exposure, calling into question the notion that these needs may only be satisfied by social experiences. Moreover, this may be taken to indicate that the domains underlying SWB are less rigid than commonly thought, and that SWB outcomes are more closely dependent on the bases and mechanisms of an experience (such as those listed at the start of the current section) rather than the designated category of that experience.

To summarise, here we suggest that nature exposure may buffer against the negative SWB outcomes of low social connectedness because it has a similar profile of SWB-related psychological and physiological effects, and because it may help to satisfy the need for connectedness.

### 1.5. Interactive Effects of Social Contact and Nature Exposure

As well as inter-domain interactions in terms of values and behaviours, a small amount of research has demonstrated that social and environmental contexts may also have interactive effects on emotional outcomes, providing initial evidence that they may interact in their influence on SWB. Staats and Hartig [87], for instance, found that natural scenery was only perceived as significantly more restorative than urban scenery when participants were instructed to imagine themselves alone; when imagining being accompanied by a close friend or relative, urban scenes were not perceived as more or less restorative than natural ones. Similarly, Johansson, Hartig and Staats [88] showed that participants only exhibited lower mood following a walk in an urban setting compared with a walk in a natural setting when they were alone; when they were accompanied by a close social tie, urban settings provided similar restoration to natural ones. In other words, these studies found that, in low-nature conditions, being in (imagined) social company was demonstrated to exert a positive effect on mood and environmental preference, while in high-nature conditions it did not have any additive effect. Although these findings are discussed by the study authors as social company detracting from the restorative potential of nature, their results may plausibly reflect the fact that both social contact and nature exposure replenish some psychological resource, which, when satiated by one, does not require further satiation by the other. Although encouraging, both studies, had relatively small sample sizes and it is hard to know how generalizable they are to non-experimental, day-to-day, contexts.

A small number of survey studies have partially addressed this and found that elevated nature exposure has greater protective effects on the physical health, mortality, stress, motivation, mental health and subjective wellbeing of people undergoing more negative events and circumstances such as socioeconomic deprivation or work stress compared with people who do not suffer these ills, suggesting a “buffering” effect [89,90,91,92,93,94,95]. Particularly salient to the present discussion are investigations into how nature exposure may mitigate adverse wellbeing outcomes in individuals subjected to social stressors. Van den Berg, Maas, Verheij and Groenewegen [96] found, for instance, that survey participants with greater greenspace coverage near their home (nearby nature) were less likely to experience adverse mental health outcomes from acute stressors (including inherently social stressors such as divorce, death of a loved one, or “interpersonal problems”) than those who resided near less greenspace. Further, Lederbogen et al. [97] observed that participants who lived, or were brought up, in more urban areas were significantly more susceptible to social stress at a neural level. Though there are many other urban-associated factors that may be responsible for this relationship (such as population density and a high level of social comparison), urbanity is correlated with naturalness, suggesting a potential role of nature exposure.

These studies provide initial evidence that an elevated level of nature in the residential environment (nearby nature) may either increase resilience to negative social situations or provide greater opportunities for recovery from them.

### 1.6. The Current Research

In sum, there is reasonable evidence to suggest that the links between social and environmental influences on mental health and wellbeing may overlap, and that nature exposure may protect individuals from some of the negative outcomes of a lack of social connectedness. The aim of the current study was to extend the work of studies into nature’s buffering effects to investigate whether it may ‘buffer’ the potential negative effect on mental health of poor social connectedness. Rather than explore the impact of acute social stressors, however, the current work focuses on the more chronic issue of social connectedness in general. Moreover, rather than explore generic levels of contact and wellbeing, our aim was, as far as possible, to explore how social contact and nature exposure over a given period of time might influence wellbeing over the same period of time, because we felt this would provide a more accurate reflection of any underlying interactive effects.

We made the following specific predictions: (a) Social connectedness, operationalised as the frequency of contact with friends and family during the last 7 days, would be positively associated with recalled subjective wellbeing over the same 7 day period; (b) Contact with nature (i.e., amount of nature visible from or near to one’s home (‘nearby nature’), and/or frequency of visits to nature during the last seven days) would be positively associated with subjective wellbeing over the same period (see [98]); and (c) Nature exposure would ‘buffer’ the effect of low social connectedness on subjective wellbeing over this period; i.e., individuals with low social connectedness during the last week would have higher subjective wellbeing last week if they nonetheless had high nature exposure during the last week compared to individuals with low social connectedness who also had low nature exposure.

## 2. Methods and Materials

### 2.1. Survey Design

A cross-sectional survey was designed to measure subjective wellbeing as the main dependent variable, social connectedness as the primary independent variable, and two types of nature exposure as potentially moderating variables. First, we carried out two pilot studies using variables adapted from the relevant literature. Convenience samples were recruited via snowballing on social media (pilot 1, N = 67; pilot 2, N = 42). Written feedback allowed us to adjust questionnaires for issues of clarity and other types of participant burden such as difficulty of recall over long time scales (guiding us to focus on a period of recall restricted to the last seven days). We examined pilot data for obvious skewness or kurtosis, and where necessary adjusted question structure, content, and response options.

The final questionnaire was distributed to a random sample of 400 UK adults via an online survey distribution platform (Survey Sampling International) which pays respondents to fill out a survey based in the Qualtrics online survey software. Demographic variables were well distributed in terms of age, sex, income, and qualification level (see Supplementary Table S1). Due to issues of reliability of data from online surveys (e.g., people not taking them seriously, or completing them over multiple sittings), data from participants with the shortest 5% (n = 20; <2 min, 9 s) and longest 5% (n = 20; >15 min, 28 s) completion times were removed *a priori* from the dataset as suggested by Malhotra [99]. An alternative method of outlier exclusion (i.e., Median Absolute Deviation; [100]) was not used because this would have resulted in asymmetric exclusion (i.e., no exclusions based on rapid completion and >10% based on slow completion). Due to missing data for one participant the final estimation sample was n = 359.

### 2.2. Measures

#### 2.2.1. Subjective Wellbeing

Subjective wellbeing was measured using the 5-item Wellbeing Index developed by the World Health Organisation (WHO-5). Used worldwide, it measures positive wellbeing but can also help identify people at risk from common mental disorders such as anxiety and depression [101,102,103,104,105]. Importantly for present concerns it has also been shown to be sensitive to greenspace access in the UK [98]. Although the WHO-5 generally asks participants to rate the extent to which they experienced various emotions over the past two weeks, the current work altered this to the last 7 days, to be consistent with other questions regarding social connectedness and nature exposure, and to fit with participant responses during piloting suggesting that they found it too hard to recall their wellbeing, social connectedness and nature exposure beyond the last seven days. Specifically, participants were presented with five statements about their emotions during the last week (“Over the last seven days…: 1. I have felt cheerful and in good spirits; 2. I have felt calm and relaxed; 3. I have felt active and vigorous; 4. I woke up feeling refreshed and rested; 5. My daily life has been filled with things that interest me”), with response scales from 0 (None of the time) to 5 (All of the time). The scale showed high internal consistency (Cronbach’s *α* = 0.91) and, as recommended, participants’ scores on the five dimensions were summed to give a score out of 25, and then multiplied by four to give a score out of 100 (with the result that raw change scores can also be considered as percentage changes). A score above 50 is considered to reflect high wellbeing, and a score below 28 is considered to indicate a risk of depression [104,105].

#### 2.2.2. Social Connectedness

Social connectedness was measured using a single item asking “Over the last 7 days, roughly how often have you met with a person or people who are important to you (i.e., friends, family) who do not live with you?” with response options: (1) 0 times; (2) 1–2 times; (3) 3–4 times; (4) 5–6 times; and, (5) 7 or more times. This measure was developed from pre-existing measures on social connectedness employed by the European Commission and by the UK Office for National Statistics (ONS) [106,107], but slightly adapted to focus on the last seven days as noted above.

#### 2.2.3. Nature Exposure

Following Natural England’s Monitor of Engagement with the Natural Environment (MENE) survey [108], participants were provided with a definition of ‘nature’ including: “*green open spaces that are dominated by nature or that have significant natural features, as well as aquatic or marine environments. These may include places such as urban parks, public gardens, woodland, farmland, beaches, canals, allotments, rivers, coasts, lakes, hills, moorland, and so on*.”

Nature exposure was measured in two ways. First, a self-assessed measure of the quantity of nature near the home or ‘Nearby nature’ was derived by collapsing the responses to two items (r = 0.78, *p* < 0.001) derived from previous studies [92,109,110,111]. The first question asked participants to rate how much nature was visible from their home, and the second asked how much nature was visible in their neighbourhood as they travelled round it, with response options from 1 (not at all natural) to 7 (completely natural). The focus on nearby nature reflects previous studies showing that the area surrounding the home is a principal source of a person’s nature exposure, and repeated exposure cumulatively influences mental wellbeing [11,21,110].

Second, participants were asked, “Over the last 7 days, how many times have you visited an area of nature for leisure or recreation?” (‘Nature visits’), with response options consistent with the social connectedness question: (1) 0 times; (2) 1–2 times; (3) 3–4 times; (4) 5–6 times; and, (5) 7 or more times. An ‘objective’ estimate of local area greenspace was not possible, since ethical approval was not available for collecting the necessary post-code level data (for this student project).

#### 2.2.4. Covariates

We identified and measured a number of confounding variables based on findings and recommendations within the literature on subjective wellbeing [6,112,113]. These included: gender (male = reference); age (≥55 years vs. 18–54 years = reference); income (≥£40,000 pa vs. <£40,000 pa = reference); educational attainment level (higher education vs. no higher education = reference); number of under 18 s in the household (≥1 vs. 0 = reference); and number of adults in the household (≥1 vs. 0 = reference).

In a relatively novel addition to the field we also included a measure of the “big five” personality traits of Openness, Conscientiousness, Extraversion, Agreeableness, and Neuroticism, i.e., the Ten-Item Personality Inventory (TIPI; [114]). These dimensions of personality (esp. extraversion) have been shown to be reliable predictors of all three of our key variables, subjective wellbeing (e.g., [4,115], social connectedness (e.g., [116]), and nature connectedness (e.g., [117]) in previous research, making it particularly important to control for when exploring whether nature exposure may moderate the relationship between social connectedness and wellbeing. See Supplementary Table S1 for frequencies and means of covariates.

### 2.3. Data Analyses

Data analysis was conducted using SPSS v24 (IBM Corporation, Armonk, NY, USA; [118]). First, we analysed zero-order correlations to identify general patterns of association and examine for multicollinearity. We then used multiple regression models to examine whether the relationship between social contact frequency and SWB depended on either of the two nature exposure variables, as stipulated by Cohen, Cohen, West and Aiken [119]. As these items were measured using different response scales we first of all standardised each scale and then multiplied the Z-scores for each together. The Z-scores for each of the main independent variables was added to the regression first, followed by the multiplication of the two Z-scores to assess the interaction effect. We then ran the same model with the addition of covariates.

In multiple regression moderation analysis, a non-significant interaction term indicates no interactive effect, with the independent variable and moderator having (potentially) simple additive effects on the dependent variable. A significant positive interaction term implies an “enhancing interaction”, in which the combined effects of the independent variable and the moderator are stronger than would be expected if they were additive. A significant negative interaction term is indicative of a “buffering interaction”, in which a higher value of the moderator diminishes the effect of the independent variable on the dependent variable [119]. Should any significant interactions emerge they can then be explored further using conditional effects analysis, which would explore, for instance, the relationship between social connectedness and SWB at different levels of nearby nature. In this model, the relationship between the independent variable (nature exposure) and the dependent variable (WHO-5 score) is explored at three values of the moderator (social connectedness): at one standard deviation above the mean (high); at the mean; and at one standard deviation below the mean (low). If our ‘nature buffering’ prediction is correct, we would expect both a significant interaction and a pattern of conditional results such that while the relationship between social connectedness and wellbeing would be strong for low nature exposure (i.e., social connectedness is very important for wellbeing with low nature exposure), it would be weaker for individuals with high nature exposure (because people with high nature exposure have an alternative pathway to wellbeing). As well as the two main regressions analysing the potentially moderating role of nearby nature and visit frequency on the whole WHO-5 scale, we also conducted binary logistic regressions on the risk of depression cut-off point (<28 vs. ≥28, see [104]), to see whether nature exposure might even moderate the relationship between social connectedness and the risk of depression, a potentially meaningful clinical outcome.

### 2.4. Ethics

Ethics were obtained from the College of Life and Environmental Sciences ethics board at the University of Exeter (ethical approval code: 2017/1513). Before taking the survey, participants were directed to a welcome page where they were informed that they could opt out of the survey at any time, and that any data they provided would be anonymous and could not be used to identify them (which is why post-code data and local area greenspace were not available).

## 3. Results

### 3.1. Descriptives

The means, standard deviations and zero-order Pearson’s correlations for our primary variables of interest are displayed in Table 1 (information for all covariates, including correlations with key variables are presented in Supplementary Table S1). Importantly, WHO-5 responses were, as predicted, significantly positively related to social contact frequency, nearby nature and nature visit frequency. The greater the level of social contact in the last seven days, the more nature near the home, and the greater the number of visits to nature in the last seven days, the higher the self-reported SWB in the last seven days. Moreover, social contact was also significantly positively correlated with both nature exposure variables.

### 3.2. Regression Analyses

Below we present results of two regressions models testing the interaction between (a) social connectedness and nearby nature (Section 3.2.1), and (b) social connectedness and nature visit frequency (Section 3.2.2).

#### 3.2.1. Social Connectedness Nearby Nature

Table 2 presents 3 models. Model 1 presents findings when just the two main predictors are entered independently, model 2 adds the interaction term, and model 3 adds the covariates. Results from model 1 support the zero-order correlations and suggest that a standard deviation increase in social connectedness and nearby nature are associated with 5.4% and 6.9% increases in WHO-5 scores respectively (as indicated by the associated B values of 5.35 and 6.91, which may be interpreted directly as percentages due to the 100-point maximum score of the WHO-5) and by themselves account for 15% of the variance in WHO-5 scores (indicated by R^2^). Model 2 adds the interaction term and, supporting predictions, it was significantly negative, suggesting a ‘buffering’ effect. The two main and interaction effects all remained significant in the fully adjusted model where extraversion and number of children in the household also emerged as significant positive predictors of WHO-5, and gender, education and openness emerged as marginally significant predictors.

**Figure 1:**
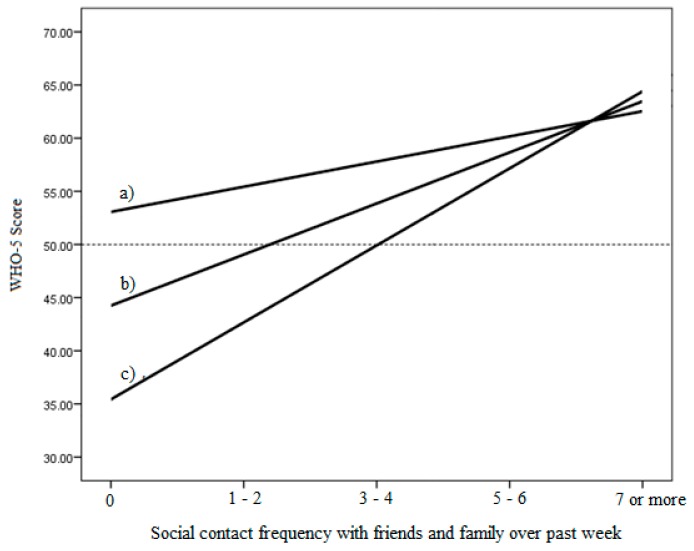
Simple slopes graph depicting conditional relationship between social contact frequency and subjective wellbeing in the last seven days: (a) at 1 standard deviation above the mean; (b) at the mean; (c) at 1 standard deviation below the mean of nearby nature. The dashed horizontal line depicts the threshold below which wellbeing is considered low [105].

To better understand the significant interaction, we conducted conditional effects analysis to calculate the un-standardised regression coefficients for social connectedness on SWB, at the mean value, at one standard deviation above the mean value and at one standard deviation below the mean value of nearby nature (using the raw connectedness scores for ease of interpretation, see Supplementary Table S2). Results, expressed as simple slopes, are plotted in Figure 1 where it can be seen that while the amount of nearby nature is very important for wellbeing among those with few social contacts in the last week, it is far less important for those with high social connectedness. Indeed, at one standard deviation above the mean levels of nearby nature, wellbeing is estimated to be above the threshold for high vs. low wellbeing (i.e., 50). Supporting the suggestion that these differences are potentially clinically meaningful, binary logistic regression analysis predicting the odds of reporting a score reflecting being at risk of depression (i.e., <28), also found a significant interaction (*p* < 0.05) once all covariates had been controlled for (Supplementary Table S3). This time the interaction term was positive, reflecting the fact that when nearby nature was high there was a lower risk of depression, and that this was especially important when social contacts were low.

#### 3.2.2. Social Connectedness*Nature Visit Frequency

**Figure 2:**
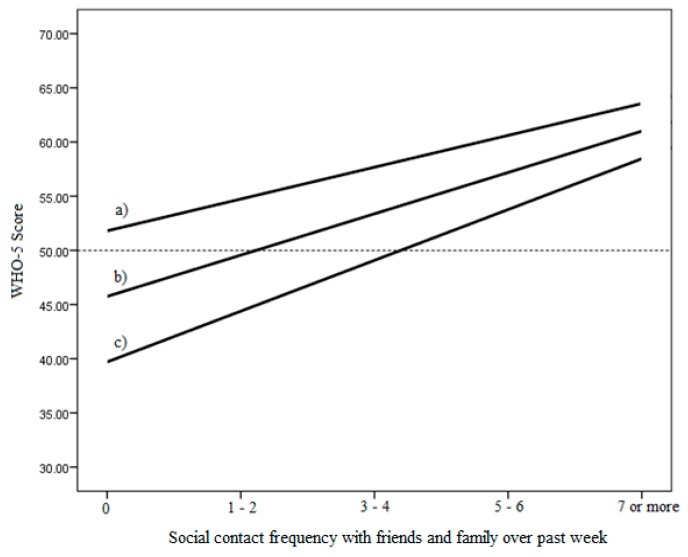
Simple slopes graph depicting conditional relationship between social contact frequency and subjective wellbeing: (a) at 1 standard deviation above the mean; (b) at the mean; (c) at 1 standard deviation below the mean of nature visit frequency. The dashed horizontal line depicts the threshold below which wellbeing is considered low [105].

Results for the regression relating to the potentially buffering effect of nature visit frequency on the relationship between social connectedness and SWB are presented in Table 3 and Figure 2. Again, social connectedness, and this time visit frequency, were associated with 4.5% and 6.5% increases in WHO-5 scores respectively (and accounted for 14% of the variance in WHO-5 scores). This time however, the interaction between them was not significant in either the unadjusted or adjusted model (where again extraversion and children in the home were also significant positive predictors). As seen in Figure 2 the three lines are more parallel suggesting mainly additive effects, although again at low levels of social contact, high nature visit frequency appears to be associated with a qualitative shift from low to high levels of wellbeing, whereas at high levels of social contact, nature visits only serve to improve already high levels of wellbeing. Replicating these results, the binary logistic regression also found no significant interaction for nature visit frequency (results available on request).

## 4. Discussion

Supporting our first two predictions, greater social connectedness in the last seven days and greater nature exposure (both in terms of nearby nature, and visits in the last week) were both associated with greater subjective wellbeing (SWB) over the same period. Moreover, in partial support of our key third prediction, nearby nature, but not visit frequency, moderated the relationship between social connectedness and SWB over the last seven days. Put simply, a relative lack of social connectedness was less important for SWB among those who lived in greener areas. Or to put it another way, nearby nature appeared to ‘buffer’ the effect of a lack of social connectedness on wellbeing, supporting and extending previous work that also suggested that living in more natural areas buffers against the adverse impacts of negative events and circumstances on stress and wellbeing (e.g., [94,95]). Importantly, this effect was replicated across both the overall WHO-5 score and a binary version which identifies a cut-off for being at risk of depression [104]. That is, high levels of nearby nature appeared to reduce the likelihood of showing signs of depression among individuals with low social connectedness. Contrary to predictions, however, intentional exposure, i.e., the number of visits made to nature in the last week, did not show a similar buffering effect, suggesting instead that socially connected individuals stand to gain roughly as much as socially isolated individuals from making more frequent visits to natural areas in terms of their SWB.

That the strength of association between our measures of nature exposure and SWB were similar in size to those between social connectedness and SWB shows just how important the local environment can be, given that social connectedness is often thought to be one of the key predictors of wellbeing [10]. The buffering effect of nearby nature on the social connectedness-SWB relationship also has important implications for the domain-based conceptualisation of human wellbeing, because it would appear that a deficit in the “social domain” of SWB may be supplanted by experiences based in the biophysical environment. This “inter-domain” interaction thus supports the possibility that both social connectedness and nature exposure may act upon shared mechanistic pathways (e.g., stress, rumination, self-esteem, etc.), and that they may be expressions of the same underlying need, i.e., the need to feel connected, whether this is manifested as being connected to other people or the natural world. Although from an evolutionary perspective this may suggest that both social and environmental factors are indicative of the perceiver’s reproductive fitness, we remain cautious about proposing evolutionary explanations given the more easily demonstrable influences of culture and individual experiences [120,121]. Clearly far more, and possibly experimental, work is needed to unpick these possibilities.

There are a number of possible reasons why we did not see an interactive effect between social connectedness and visit frequency. One possibility is due to a restricted data range. Specifically, although nearby nature had a good distribution across the 1–7 scale, approximately a third of participants reported not visiting the natural environment at all in the last seven days and only 25% reported visiting more than once or twice. Thus although, in some ways, nature visit frequency is a more directly comparable measure of ‘contact’ to the frequency of social contact experiences, it has a more restricted range than nearby nature, which people may still experience indirectly (e.g., through window views, e.g., [11]). It may also be that visiting nature is not as “clean” a measure of nature exposure as nearby nature in that it also (usually) involves other things which promote wellbeing but which were not included in the current analysis such as some form of physical activity [15,16,122,123]. Although we are unsure exactly why the associations between the measures differed here, we nonetheless believe it is important to test both, and other, operationalisations of nature exposure in future work to help us gain a better understanding of how different measures of nature exposure reflect different types of experience.

A relatively novel aspect of the current work was its inclusion of personality among the set of covariates. This was important because it meant that an aspect of an individual that might influence both their desire for contact with others, and their desire for contact with nature, was controlled for, reducing the possibility that any interaction was due to an unknown third factor (in this case personality). Reflecting earlier work [4,115], extraversion was positively related to SWB, and importantly for current purposes it was also significantly positively related to both measures of nature exposure, although somewhat surprisingly was non-significantly positively related to social connectedness (see Supplementary Table S1). In other words, by including extraversion in our covariate set we were able to reduce the potential problem that extraverts are both happier and more likely to report higher nature exposure, and thus any association between SWB and nature exposure may simply be due to their shared variance with extraversion. We suggest that future studies exploring nature exposure and health and wellbeing outcomes may also want to attempt to control for this often unobserved yet stable source of heterogeneity. 

Of course, we also recognise several limitations with the current study. First, the sampling methods could be improved. Due to restrictions on budget, time and resources associated with the current MSc project, the sample size was small and was not weighted to be population representative. As such, the results are not yet generalizable beyond the current sample. Future research might therefore want to explore whether the current findings might replicate across larger, more population representative surveys such as the Monitor for Engagement with the Natural Environment [108]. Second, even though personality was included, the cross-sectional methodology still prohibits causal inference, although it would be hard to explain the interactive effect between social connectedness and nearby nature though a reverse direction account from wellbeing to the moderating effect of nature exposure on social contact. Third, all variables were subject to the limitations of self-report data, and we were unfortunately unable to collect objective nearby nature in the current study. Mitigating this issue however, is the suggestion that subjective measurements of nature exposure may be of equal or greater importance than “objective” ones in any case [124]. Nevertheless, further longitudinal survey data (e.g., [52,53]) would enable future research to explore how fluctuations in social connectedness and nature exposure for the same individuals are related to fluctuations in mental health and SWB over time and experimental studies, perhaps similar to those by Hartig and colleagues [87,88], would enable a more targeted exploration of our core hypothesis. Finally, we also recognise that several potentially important covariates were not included in the survey, including employment status, physical health, and physical activity level, which can all have strong influences on mental wellbeing [6,125]. As noted above the lack of physical activity data, and thus the ability to control for it, may in part explain our inability to find a moderating effect of nature visit frequency on the social connectedness-SWB relationship.

Importantly, we wish to stress that the ways we operationalised SWB, social connectedness and nature exposure may not reflect individuals’ entire experiences of the phenomena we wished to measure. In particular, because participants only reported on their experiences in the seven days before they took the survey, it would be unwise to suggest that our results generalise to related phenomena in the longer-term, such as extended periods of social isolation.

These limitations notwithstanding, results have potential implications for policy and practice in dealing with the issues of social isolation and population wellbeing. Specifically, our data suggest that the greening or naturalisation of residential environments may provide a buffer against social isolation, in a similar way to other stressors. In the UK, for instance, those most at risk of social isolation tend to be older individuals, ethnic minorities, and those living in more deprived areas; the exact same groups who tend to have lower than average contact with nature [98,126,127,128]. Consequently, increasing the quantity of nature in neighbourhoods dominated by these and other isolated population segments is a potentially important public health good. At the individual-level, our results suggest that people who are socially disconnected, for instance when moving to a new area, may avoid the worst effects of social isolation if they move to a more natural location. Finally, our results suggest that nature visit frequency may be beneficial for SWB at various levels of social connectedness, and so even the most socially connected person may still stand to benefit from actively going out to experience nature.

## 5. Conclusions

Our study corroborates previous findings that social connectedness and nature exposure are influential in mental health and wellbeing, reaffirming intuitions and empirical findings that contact with nature and contact with other people are both beneficial in their own right. Furthermore, we find that individuals who are less socially connected are more likely to report positive subjective wellbeing and less likely to suffer from depression when they have greater nature exposure, suggesting that visiting and living amongst nature may help to improve wellbeing and avoid mental health disorders in individuals who are suffering from social isolation, at least temporarily.

We also find partial support for our novel hypothesis that nature exposure moderates the relationship between social connectedness and mental health and wellbeing. More nearby nature around the home and neighbourhood appears to “buffer” against the adverse wellbeing outcomes of low social connectedness, but does not improve wellbeing in socially connected people. This may reflect a shared psychological resource that both social connectedness and nearby nature act upon, which we hypothesise to be a general feeling of connection. Further cross-sectional, longitudinal and experimental studies exploring these issues are now needed to better understand the complex interplay of our relationships to other people and the natural world for our mental health and wellbeing.

## Figures and Tables

**Table 1:** Pearson’s zero-order correlations, means and standard deviations of the main variables of interest.

	1	2	3	4
1. Social contact frequency	--			
2. Nearby nature	0.13 **	--		
3. Nature visit frequency	0.28 ***	0.28 ***	--	
4. WHO-5 score	0.27 ***	0.32 ***	0.32 ***	--
*Mean*	2.60	3.97	2.06	51.56
*SD*	1.18	1.58	1.04	23.38

** *p* < 0.01; *** *p* < 0.001.

**Table 2:** Nearby nature*Social contact. Results of multiple regression predicting subjective wellbeing, displaying: unstandardised regression coefficients (B); standard error (SE); standardised regression coefficients (β); and, significance (*p*) of coefficients.

	Model 1			Model 2			Model 3		
	B (SE)	β	*p*	B (SE)	β	*p*	B (SE)	β	*p*
*Key variables*									
ZSocial contact	5.35 (1.13)	0.23	<0.001	5.64 (1.13)	0.25	<0.001	5.49 (1.12)	0.24	<0.001
ZNearby nature	6.91 (1.16)	0.29	<0.001	6.84 (1.15)	0.29	<0.001	5.24 (1.22)	0.22	<0.001
ZSocial contact x ZNearby nature				−2.50 (1.16)	−0.11	<0.05	−2.87 (1.15)	−0.12	0.013
*Demographics*									
Age (ref = under 55 years)							−1.06 (2.89)	−0.02	0.713
Gender (ref = male)							−4.06 (2.26)	−0.09	0.073
Income (ref = under £40,000 pa)							−2.02 (2.84)	−0.04	0.477
Education (ref = no university education)							4.67 (2.39)	0.10	0.051
Over 18 s in household (ref = none)							−1.13 (3.20)	−0.02	0.725
Under 18 s in household (ref = none)							6.34 (2.54)	0.13	0.013
*Personality*									
Extraversion							3.71 (1.38)	0.15	0.008
Agreeableness							−0.33 (1.26)	−0.02	0.791
Conscientiousness							0.68 (1.31)	0.03	0.606
Neuroticism							−1.28 (1.39)	−0.05	0.355
Openness to experience							2.16 (1.21)	0.10	0.075
N	359			359			359		
R^2^Δ	0.16 ***			0.01 *			0.08 ***		
R^2^adj	0.15			0.16			0.22		

* *p* < 0.05; *** *p* < 0.001.

**Table 3:** Nature visit frequency*social contact. Results of multiple regression predicting subjective wellbeing, displaying: unstandardised regression coefficients (B); standard error (SE); standardised regression coefficients (β); and, significance (*p*) of coefficients.

	Model 1			Model 2			Model 3		
	B (SE)	β	*p*	B (SE)	β	*p*	B (SE)		*p*
*Key variables*									
ZSocial contact	4.47 (1.18)	0.19	<0.001	4.49 (1.18)	0.20	<0.001	4.42 (1.17)	0.19	<0.001
ZNature visit frequency	6.51 (1.23)	0.27	<0.001	6.62 (1.27)	0.27	<0.001	4.91 (1.30)	0.20	<0.001
ZSocial contact x ZVisit frequency				−0.40 (1.06)	−0.02	0.70	−1.04 (1.05)	−0.05	0.320
*Demographics*									
Age (ref = under 55 years)							1.63 (2.93)	0.03	0.578
Gender (ref = male)							−3.19 (2.30)	−0.07	0.166
Income (ref = under £40,000 pa)							−0.88 (2.87)	−0.02	0.761
Education (ref = no university education)							3.47 (2.42)	0.07	0.153
Over 18 s in household (ref = none)							−0.13 (3.23)	−0.00	0.967
Under 18 s in household (ref = none)							5.55 (2.58)	0.11	0.032
*Personality*									
Extraversion							5.23 (1.37)	0.22	<0.001
Agreeableness							−1.64 (1.27)	−0.07	0.198
Conscientiousness							1.45 (1.30)	0.06	0.266
Neuroticism							−1.01 (1.41)	−0.04	0.474
Openness to experience							1.64 (1.24)	0.07	0.188
N	359			359			359		
R^2^Δ	0.14 ***			0.00			0.08 ***		
R^2^adj	0.14			0.13			0.19		

*** *p* < 0.001.

## Supplementary Materials

The following are available online at http://www.mdpi.com/1660-4601/15/6/1238/s1. Table S1: Descriptive data for all covariates; Table S2: Unstandardised conditional effects of social contact frequency on mental wellbeing at values of the moderators; Table S3: Binary logistic regression predicting the Odds Ratio (OR) of reporting depression.
