# Supplementary material for: Nearby Nature ‘Buffers’ the Effect of Low Social Connectedness on Adult Subjective Wellbeing over the Last 7 Days
Source: Int J Environ Res Public Health. 2018 Jun 12;15(6):1238. doi: 10.3390/ijerph15061238 (PMC6025411; doi:10.3390/ijerph15061238)
Supplement: Supplementary file 1 [file ijerph-15-01238-s001.pdf]

Supplementary Materials

Table S1: Descriptive data for all covariates

	**Descriptives**		**Correlation with WHO5**
Binary variables	N	(%)	r_{pb}
Scale variables	Mean	(SD)	r
Age			-.04
< 55yrs	272	75.6	
≥ 55yrs	88	24.4	
Gender			-.12*
Males	190	52.8	
Females	170	47.2	
Income			.06
< £40k p.a.	277	76.9	
≥ £40k p.a.	83	23.1	
Education level			.12*
No university education	201	55.8	
Bachelor's degree or higher	159	44.2	
Over 18s in household			.02
None	53	14.7	
≥ 1	307	85.3	
Under 18s in household			.12*
None	228	63.3	
≥ 1	132	36.7	
Personality			
Extraversion	4.33	(0.97)	.29***
Agreeableness	4.61	(1.04)	.07
Conscientiousness	4.14	(1.03)	.16**
Neuroticism	4.29	(0.97)	.08
Openness	4.41	(1.03)	.31***

* $p < .05$; ** $p < .01$; *** $p < .001$

Table S2: Unstandardised conditional effects of social contact frequency on mental wellbeing at values of the moderators.

	**Moderator**					
	**Nearby nature**			**Nature visit frequency**		
Value of moderator	**B**	**se**	**p**	**B**	**se**	**p**
One SD below mean	7.25	1.50	< . 001	4.69	1.38	< .001
At the mean	4.81	0.97	< .001	3.81	1.00	< .001
One SD above mean	2.37	1.25	.06	2.94	1.27	< .05

Table S3: Binary logistic regression predicting the Odds Ratio (OR) of reporting depression.

	Model 1			Model 2			Model 3		
	OR	95% CIs	p	OR	95% CIs	p	OR	95% CIs	p
Key variables									
ZSocial contact	0.77	0.57 – 1.04	.09	0.77	0.56 – 1.05	.09	0.75	0.54 - 1.05	.090
ZNearby nature	0.73	0.54 – 0.97	.03	0.7	0.57 – 1.03	.07	0.80	0.58 – 1.12	.191
ZSocial contact x ZNearby nature				1.29	0.95 – 1.76	.10	1.40	1.00 - 1.96	.048
Demographics									
Age (ref = under 55yrs)							1.77	0.85 – 3.68	.125
Gender (ref = male)							1.44	0.78 – 2.67	.247
Income (ref = under £40,000 pa)							1.58	0.72 – 3.47	.259
Education (ref = no university education)							0.63	0.32 – 1.22	.170
Over 18s in household (ref = none)							0.63	0.28 - 1.37	.242
Under 18s in household (ref = none)							0.53	0.25 – 1.12	.096
Personality									
Extraversion							0.67	0.46 – 0.98	.040
Agreeableness							0.93	0.67 – 1.30	.675
Conscientiousness							0.96	0.68 – 1.36	.818
Neuroticism							1.41	0.97 – 2.04	.072
Openness to experience							0.92	0.65 – 1.30	.629
N		359			359			359	
-2 Log likelihood		312.40			309.74			285.73	
Pseudo R^2 (Nagelkerke)		0.04			0.05			.16	
